# Characterization of bone marrow derived mesenchymal stem cells in suspension

**DOI:** 10.1186/scrt131

**Published:** 2012-10-19

**Authors:** Kentaro Akiyama, Yong-Ouk You, Takayoshi Yamaza, Chider Chen, Liang Tang, Yan Jin, Xiao-Dong Chen, Stan Gronthos, Songtao Shi

**Affiliations:** 1 Center for Craniofacial Molecular Biology, University of Southern California, 2250 Alcazar Street, CSA 103, Los Angeles, CA 90033, USA; 2 Department of Oral Rehabilitation and Regenerative Medicine, Okayama University Graduate School of Medicine, Dentistry, and Pharmaceutical Science, 2-5-1 Shikata-cho, Kita-ku, Okayama 700-8525, Japan; 3 Department of Molecular Cell Biology and Oral Anatomy, Kyushu University Graduate School of Dental Science, Fukuoka 812-8582, Japan; 4 Research and Development Center for Tissue Engineering, Fourth Military Medical University, Xi'an, Shanxi, China; 5 Division of Research, Department of Comprehensive Dentistry, The University of Texas Health Science Center at San Antonio, 7703 Floyd Curl Drive, San Antonio, Texas 78229-3900, USA; 6 Mesenchymal Stem Cell Group, Department of Haematology, Institute of Medical and Veterinary Science/ Hanson Institute, Adelaide 5000, South Australia, Australia

## Abstract

**Introduction:**

Bone marrow mesenchymal stem cells (BMMSCs) are a heterogeneous population of postnatal precursor cells with the capacity of adhering to culture dishes generating colony-forming unit-fibroblasts (CFU-F). Here we identify a new subset of BMMSCs that fail to adhere to plastic culture dishes and remain in culture suspension (S-BMMSCs).

**Methods:**

To catch S-BMMSCs, we used BMMSCs-produced extracellular cell matrix (ECM)-coated dishes. Isolated S-BMMSCs were analyzed by *in vitro *stem cell analysis approaches, including flow cytometry, inductive multiple differentiation, western blot and *in vivo *implantation to assess the bone regeneration ability of S-BMMSCs. Furthermore, we performed systemic S-BMMSCs transplantation to treat systemic lupus erythematosus (SLE)-like MRL/*lpr *mice.

**Results:**

S-BMMSCs are capable of adhering to ECM-coated dishes and showing mesenchymal stem cell characteristics with distinction from hematopoietic cells as evidenced by co-expression of CD73 or Oct-4 with CD34, forming a single colony cluster on ECM, and failure to differentiate into hematopoietic cell lineage. Moreover, we found that culture-expanded S-BMMSCs exhibited significantly increased immunomodulatory capacities *in vitro *and an efficacious treatment for SLE-like MRL/*lpr *mice by rebalancing regulatory T cells (Tregs) and T helper 17 cells (Th17) through high NO production.

**Conclusions:**

These data suggest that it is feasible to improve immunotherapy by identifying a new subset BMMSCs.

## Introduction

Bone marrow mesenchymal stem cells (BMMSCs) are hierarchical postnatal stem/progenitor cells capable of self-renewing and differentiating into osteoblasts, chondrocytes, adipocytes, and neural cells [1,2]. BMMSCs express a unique surface molecule profile, including expression of STRO-1, CD29, CD73, CD90, CD105, CD146, Octamer-4 (Oct4), and stage-specific embryonic antigen-4 (SSEA4) [3,4]. It is generally believed that BMMSCs are negative for hematopoietic cell markers such as CD14 and CD34 [5-13]. BMMSCs have been widely used for tissue engineering [14-16]. Recently, a growing body of evidence has indicated that BMMSCs produce a variety of cytokines and display profound immunomodulatory properties [17-19], perhaps by inhibiting the proliferation and function of several major immune cells, such as natural killer cells, dendritic cells, and T and B lymphocytes [17-20]. These unique properties make BMMSCs of great interest for clinical applications in the treatment of different immune disorders [17,21-24].

BMMSCs are thought to be derived from the bone marrow stromal compartment, initially appearing as adherent, single colony clusters (colony-forming unit-fibroblasts [CFU-F]), and subsequently proliferating on culture dishes [25]. To date, the CFU-F assay has been considered one of the gold standards for determining the incidence of clonogenic BMMSC [26,27]. Since BMMSC are a heterogeneous population of stem cells, it is critical to identify whether BMMSC contain unique cell subsets with distinctive functions, analogous to the hematopoietic stem/progenitor cell system. In this study, we identified a subset of mouse BMMSCs in culture suspension and determined their immunomodulatory characteristics.

## Materials and methods

### Animals

Female C3H/HeJ, C57BL/6J, and C3MRL-Fas^lpr^/J mice were purchased from Jackson Laboratory (Bar Harbor, ME, USA). Female immunocompromised mice (Beige *nude*/*nude *XIDIII) were purchased from Harlan (Indianapolis, IN, USA). All animal experiments were performed under the institutionally approved protocols for the use of animal research (USC #10874 and 10941).

### Antibodies

Anti Oct4, SSEA4, Runx2, OCN, active β catenin and β catenin were purchased from Millipore (Billerica, MA, USA). Anti alkaline phosphatase (ALP) antibody was purchased from Abcam (Cambridge, MA, USA). Anti Sca-1-PE, CD34-PE, CD34-FITC, CD45-PE, CD73-PE, CD4-PerCP, CD8-FITC, CD25-APC, CD3ε and CD28 antibodies were purchased from BD Bioscience (San Jose, CA, USA). Anti Foxp3-PE, IL17-PE, and IFNγ-APC antibodies were purchased from eBioscience (San Diego, CA, USA). Unconjugated anti CD34, CD73, and CD105, NOS2 were purchased from Santa Cruz Biosciences (Santa Cruz, CA, USA). Anti β actin antibody was purchased from Sigma (St. Louis, MO, USA).

### Isolation of mouse bone marrow mesenchymal stem cells (BMMSCs)

The single suspension of bone marrow derived all nucleated cells (ANCs) from femurs and tibias were seeded at a density of 15 × 10^6 ^into 100 mm culture dishes (Corning, NY, USA) at 37°C and 5% CO2. Non-adherent cells were removed after two days and attached cells were maintained for 16 days in alpha minimum essential medium (α-MEM, Invitrogen, Grand Island, NY, USA) supplemented with 20% fetal bovine serum (FBS, Equitech-bio, Kerrville, TX, USA), 2 mM L-glutamine, 55 μM 2-mercaptoethanol, 100 U/ml penicillin, and 100 μg/ml streptomycin (Invitrogen). Colony-forming attached cells were passed once for further experimental use.

### Preparation of Extracellular Matrix (ECM) coated dishes

ECM coated dishes were prepared as described previously [28]. Briefly, 100% confluence of BMMSCs was cultured in medium with 100 nM L-ascorbic acid phosphate (Wako Pure Chemical, Richmond, VA, USA). After two weeks, cultures were washed with PBS and incubated with 0.005% Triton X-100 (Sigma) for 15 minutes at room temperature to remove cells. The ECM was treated with DNase I (100 units/ml; Sigma) for 1 hour at 37°C. The ECM was washed with PBS three times and stored in 2 ml of PBS containing 100 U/ml penicillin, 100 μg/ml streptomycin and 0.25 μg/ml fungizone (Invitrogen) at 4°C.

### Isolation of BMMSCs in culture suspension (S-BMMSCs)

Bone marrow-derived ANCs (15 × 10^6^) were seeded into 100 mm culture dishes and cultured for two days. The culture supernatant with floating cells was collected and centrifuged to obtain putative non-attached BMMSCs. The cells were re-seeded at indicated numbers on ECM-coated dishes. After 2 days, the floating cells in the cultures were removed with PBS and the attached cells on ECM were maintained for an additional 14 days. Colony-forming attached cells were passed once and sub-cultured on regular plastic culture dishes for further experiments. For some stem cell characterization analyses, we collected SSEA4 positive S-BMMSCs using the MACS magnetic separation system (Milteny Biotech, Auburn, CA, USA) and expanded in the cultures.

### Colony forming unit-fibroblastic (CFU-F) assay

One million cells of ANCs from bone marrow were seeded on a T-25 cell culture flask (Nunc, Rochester, NY, USA). After 16 days, the cultures were washed with PBS and stained with 1% toluidine blue solution in 2% paraformaldehyde (PFA). A cell cluster that had more than 50 cells was counted as a colony under microscopy. The colony number was counted in five independent samples per each experimental group.

### Cell proliferation assay

The proliferation of BMMSCs and S-BMMSCs was performed using the bromodeoxyuridine (BrdU) incorporation assay. Each cell population (1 × 10^4 ^cells/well) was seeded on two-well chamber slides (Nunc) and cultured for two to three days. The cultures were incubated with BrdU solution (1:100) (Invitrogen) for 20 hours, and stained with a BrdU staining kit (Invitrogen). BrdU-positive and total cell numbers were counted in ten images per subject. The BrdU assay was repeated in five independent samples for each experimental group.

### Population doubling assay

A total of 0.5 × 10^6 ^cells of BMMSCs and S-BMMSCs was seeded on 60 mm culture dishes at the first passage. Upon reaching confluence, the cells were passaged at the same cell density. The population doubling was calculated at every passage according to the equation: log_2 _(number of harvested cells/number of seeded cells). The finite population doublings were determined by cumulative addition of total numbers generated from each passage until the cells ceased dividing.

### Flow cytometric analysis of mesenchymal stem cell surface molecules

BMMSCs or S-BMMSCs (0.2 × 10^6 ^cells) were incubated with 1 µg of R-Phycoerythrin (PE). (PE)-conjugated antibodies or isotype-matched control immunoglobulin Gs (IgGs) (Southern Biotech, Birmingham, AL, USA) at 4°C for 45 minutes. Samples were analyzed by a fluorescence-activated cell sorting (FACS)^Calibur ^flow cytometer (BD Bioscience). For dual color analysis, the cells were treated with PE-conjugated and fluorescein isothiocyanate (FITC)-conjugated antibodies or isotype-matched control IgGs (1 µg each). The cells were analyzed on FACS^Calibur ^(BD Bioscience).

### Immunofluorescent microscopy

The cells subcultured on eight-well chamber slides (Nunc) (2 × 10^3^/well) were fixed with 4% PFA. The samples were incubated with the specific or isotype-matched mouse antibodies (1:200) overnight at 4°C, and treated with Rhodamine-conjugated secondary antibodies (1:400, Jackson ImmunoResearch, West Grove, PA, USA; Southern Biotechnology, Birmingham, AL, USA). Finally, chamber slides were mounted using Vectashield mounting medium containing 4', 6-diamidino-2-phenylindole (DAPI) (Vector Laboratories, Burlingame, CA, USA).

### *In vivo *bone formation assay

A total of 4.0 × 10^6 ^cells was mixed with hydroxyapatite/tricalcium phosphate (HA/TCP) ceramic powders (40 mg, Zimmer Inc., Warsaw, IN, USA) and subcutaneously transplanted into eight-week-old immunocompromised mice. After eight weeks, the transplants were harvested, fixed in 4% PFA and then decalcified with 5% ethylenediaminetetraacetic acid (EDTA; pH 7.4), followed by paraffin embedding. The paraffin sections were stained with H & E and analyzed by an NIH Image-J. The newly-formed mineralized tissue area from five fields was calculated and shown as a percentage to total tissue area.

### *In vitro *osteogenic differentiation assay

BMMSCs and S-BMMSCs were cultured under osteogenic culture conditions containing 2 mM β-glycerophosphate (Sigma), 100 μM L-ascorbic acid 2-phosphate and 10 nM dexamethasone (Sigma). After induction, the cultures were stained with alizarin red or alkaline phosphatase.

### *In vitro *adipogenic differentiation assay

For adipogenic induction, 500 nM isobutylmethylxanthine, 60 μM indomethacin, 500 nM hydrocortisone, 10 μg/ml insulin (Sigma), 100 nM L-ascorbic acid phosphate were added to the culture medium. After 10 days, the cultured cells were stained with Oil Red-O and positive cells were quantified by using an NIH Image-J. Total RNA was also isolated from cultures after 10 days induction for further experiments.

### *In vitro *chondrogenic differentiation assay

For chondrogenic induction, 1 × 10^6 ^cell pellets were cultured under chondrogenic medium containing 15% FBS, 1% ITS (BD), 100 nM dexamethasone, 2 mM pyruvate (SIGMA), and 10 ng/ml transforming growth factor beta 1 (TGFβ1) in (D)MEM (Invitrogen) for threeweeks. Cell pellets were harvested at three weeks post induction, fixed overnight with 4% PFA and then, sections were prepared for staining.

### Reverse transcriptase polymerase chain reaction *(*RT-PCR*) *analysis

Extraction of total RNA and RT-PCR were performed according to standard procedures. Primer information is described in Additional materials and methods [see Additional file 1].

### Western blotting analysis

A total of 20 µg of protein was used and SDS-PAGE and western blotting were performed according to standard procedures. Detailed procedures are described in Additional materials and methods [see Additional file 1]. β-actin on the same membrane served as the loading control.

### Hematopoietic differentiation of BMMSCs and S-BMMSCs

BMMSCs and S-BMMSCs were cultured onto 35 mm low attach culture dishes (2 × 10^4^/dish, STEMCELL Technologies, Vancouver, BC, V5Z 1B3, Canada) under hematopoietic differentiation medium (STEMCELL Technologies) with or without erythropoietin (EPO; 3 U/mL) for seven days. Whole bone marrow cells and linage negative bone marrow cells (Linage-cells) were used as positive controls. The results are representative of five independent experiments.

### Inhibitor treatment

S-BMMSCs and BMMSCs were treated with 1 mM L-NG-monomethyl-arginine (L-NMMA) (Cayman Chemical, Ann Arbor, MI, USA) or 0.2 mM 1400 W (Cayman Chemical) to inhibit total nitric oxide synthase (NOS) or inducible nitric oxide synthase (iNOS), respectively.

### Measurement of nitric oxide production

BMMSCs (0.2 × 10^6^/well) were cultured on 24-well plates with or without cytokines (IFNγ, 25 ng/ml; IL-1β, 5 ng/ml, R&D Systems, Minneapolis, MN, USA) and chemicals (L-NMMA, 1 mM; 1400 W, 0.2 mM) at the indicated concentration and days. The supernatant from each culture was collected and nitric oxide concentration measured using a Total Nitric Oxide and Nitrate/Nitrite Parameter Assay kit (R&D Systems) according to the manufacturer's instruction.

### Cell apoptosis and cell survival assay

The transwell system (Corning) was used for co-culture experiments. A total of 0.2 × 10^6 ^of S-BMMSCs or BMMSCs was seeded on each lower chamber. Activated spleen cells (1 × 10^6^/chamber), which were pre-stimulated with plate-bound anti CD3ε antibody (3 µg/ml) and soluble anti CD28 antibody (2 µg/ml) for two days, were loaded in the upper chambers. Both chambers were filled with a complete medium containing (D)MEM (Lonza, CH-4002 Basel, Switzerland) with 10% heat-inactivated FBS, 50 µM 2-mercaptoethanol, 10 mM HEPES, 1 mM sodium pyruvate (Sigma), 1% non-essential amino acid (Cambrex, East Rutherford, NJ, USA), 2 mM L-glutamine, 100 U/ml penicillin and 100 mg/ml streptomycin. To measure the spleen cells viability, cell counting kit-8 (Dojindo Molecular Technologies, Rockville, MD, USA) was used. For apoptosis of spleen cells analyses, Annexin V-PE apoptosis detection kits I (BD Bioscience) were used and analyzed on FACS^Calibur ^(BD Bioscience).

### *In vitro *CD4^+^CD25^+^Foxp3^+^Tregs and Th17 induction

CD4^+^CD25^- ^T-lymphocytes (1 × 10^6^/well), collected using a CD4^+^CD25^+ ^Treg isolation kit (Miltenyi Biotec), were pre-stimulated with plate-bound anti CD3ε antibody (3 µg/ml) and soluble anti CD28 antibody (2 µg/ml) for two days. These activated T-lymphocytes were loaded on 0.2 × 10^6 ^BMMSCs or S-BMMSCs cultures with recombinant human TFGβ1 (2 ng/ml) (R&D Systems) and recombinant mouse IL2 (2 ng/ml) (R&D Systems). For Th17 induction, recombinant human TFGβ1 (2 ng/ml) and recombinant mouse IL6 (50 ng/ml) (Biolegend, San Diego, CA, USA) were added. After three days, cells in suspension were collected and stained with anti CD4-PerCP, anti CD8a-FITC, anti CD25-APC antibodies (each 1 µg) for 45 minutes on ice under dark conditions. The cells were then stained with anti Foxp3-PE antibody (1 µg) using a Foxp3 staining buffer kit (eBioscience) for cell fixation and permeabilization. For Th17, cells in suspension were stained with anti CD4-FITC (1µg, Biolegend) for 45 minutes on ice under dark conditions followed by intercellular staining with anti-IL 17 antibody (1µg, Biolegend) using a Foxp3 staining buffer kit. The cells were analyzed on FACS^Calibur^.

### Allogenic mouse S-BMMSC transplantation into MRL/lpr mice

Under general anesthesia, C3H/HeJ-derived BMMSCs, S-BMMSCs, L-NMMA pre-treated BMMSCs (1 mM for five days), or CD34^+^/CD73^+ ^double sorted cells (0.1 × 10^6 ^cells/10 g body weight) were infused into MRL/*lpr *mice via the tail vein at 10 weeks of age (n = 6 each group). In the control group, MRL/*lpr *mice received PBS (n = 5). All mice were sacrificed at two weeks post transplantation for further analysis. The protein concentration in urine was measured using a Bio-Rad Protein Assay (Bio-Rad, Hercules, CA, USA).

### Measurement of autoantibodies, albumin, soluble runt-related NF-κB ligand (sRANKL) and C-terminal telopeptides of type I collagen (CTX)

Peripheral blood serum samples were collected from mice. Autoantibodies, sRANKL and CTX were analyzed by ELISA using commercially available kits (anti-dsDNA antibodies and ANA; alpha diagnostics, albumin and sRANKL; R&D Systems, CTX; Nordic Bioscience Diagnostics, Herlev, Rigion Hovedstaden, Denmark) according to their manufactures' instructions. The results were averaged in each group. The intra-group differences were calculated between the mean values.

### Flow cytometric analysis of Tregs and Th17 cells

To detect Tregs, peripheral blood mononuclear cells (PBMNCs) (1 × 10^6^) were treated with PerCP-conjugated anti-CD4, FITC-conjugated anti-CD8a, APC-conjugated anti-CD25 antibodies, and stained with R-PE-conjugated anti-Foxp3 antibody using a Foxp3 staining buffer kit (eBioscience). To measure Th17 cells, PBMNCs (1 × 10^6^) were incubated with PerCP-conjugated anti-CD4, FITC-conjugated anti-CD8a, followed by treatment with R-PE-conjugated anti-IL-17 and APC-conjugated anti-IFNγ antibodies using a Foxp3 staining buffer kit. The cells were then analyzed on FACS^Calibur ^.

### Statistics

Student's t-test was used to analyze statistical difference. *P *values less than 0.05 were considered significant.

## Results

### A subset of BMMSCs lacks the ability to adhere to plastic culture dishes (S-BMMSCs) but attaches to extracellular cell matrix (ECM)-coated culture dishes

**Figure 1:**
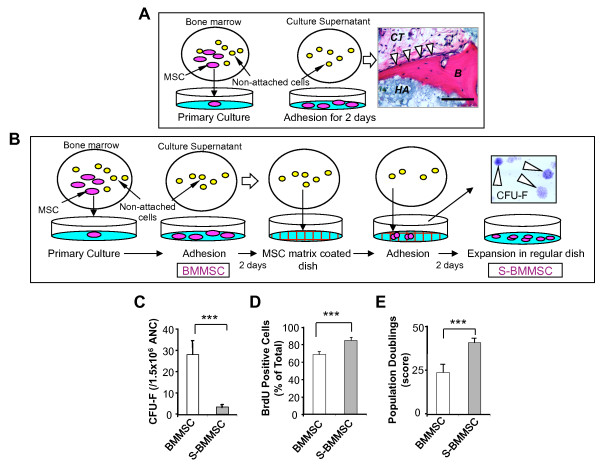
**Identification of suspension BMMSCs (S-BMMSCs)**. (**A**) Hypothetical model indicates that bone marrow all nucleated cells (ANCs) were seeded at 15 × 10^6 ^into 100 mm culture dishes and incubated for two days at 37°C with 5% CO2, and subsequently non-attached cells from culture suspension were transplanted into immunocompromised mice subcutaneously using hydroxyapatite tricalcium phosphate (HA) as a carrier for eight weeks. Newly formed bone (B) by osteoblasts (arrow heads) and associated connective tissue (C) were detected in this non-attached cell transplants by H & E staining. Bar = 100 μm. (**B**) Hypothetical model of isolating S-BMMSCs. BMMSCs usually attach on culture dishes within two days; however, a small portion of BMMSCs in ANCs failed to attach to the dishes and remained in the suspension. The suspensions containing putative non-attached BMMSCs were collected and transferred to the extracellular matrix (ECM) coated dish with generating single colony clusters (CFU-F). These ECM-attached BMMSCs (S-BMMSCs) were sub-cultured on regular plastic culture dishes for additional experiments. (**C**) The number of plastic attached CFU-F from ANCs (1.5 × 10^6 ^cells) is more than seven-fold higher than that derived from BMMSC-ECM adherent S-BMMSCs. (**D**) Proliferation rates of S-BMMSCs and BMMSCs were assessed by BrdU incorporation for 24 hours. The percentage of positive cells is significantly increased in S-BMMSCs when compared to BMMSCs. (**E**) S-BMMSCs exhibit a significant increase in population doublings when compared to BMMSCs. The results are representative of five independent experiments. Scale bars = 50 μm. ***P <0.001. The graph bar represents mean ± SD. BMMSCs, bone marrow mesenchymal stem cells; BrdU, bromodeoxyuridine; S-BMMSCs, BMMSCs in suspension; SD, standard deviation.

To determine whether a subset of BMMSCs remain in culture suspension, ANCs (15 × 10^6 ^cells) from bone marrow were plated onto regular plastic culture dishes for two days and all non-attached cells were subsequently transplanted into immunocompromised mice subcutaneously using HA/TCP as a carrier. At eight weeks post-transplantation, newly formed bone was identified in the transplants by H & E staining (Figure 1A), suggesting that the BMMSC culture suspension may contain cells with a capacity to differentiate into bone forming cells. *In vitro *studies indicated that ECM produced by culture-expanded BMMSCs (BMMSC-ECM) could capture higher numbers of CFU-Fs when compared to plastic cultures [see Additional file 1, Figure S1] [28]. Thus, we collected culture supernatant with floating cells at two days post CFU-F culture and re-loaded it onto BMMSC-ECM-coated dishes (Figure 1B). A subset of BMMSCs in the suspension (S-BMMSCs) was able to adhere to the BMMSC-ECM and form CFU-F (Figure 1B), at a lower incidence compared to the number of CFU-F generated from regular BMMSCs (Figure 1C). In order to characterize the stem cell properties of S-BMMSCs, we collected SSEA4-positive S-BMMSCs and assessed their proliferation rate by BrdU incorporation. We found that S-BMMSCs had a significantly elevated BrdU uptake rate compared to regular BMMSCs (Figure 1D). In addition, we used a continuous cell culture assay to indicate that SSEA4-positive S-BMMSCs acquired a significantly increased number of population doublings (Figure 1E). These data imply that S-BMMSCs are distinct from regular BMMSCs in terms of attachment, proliferation, and self-renewal.

**Figure 2:**
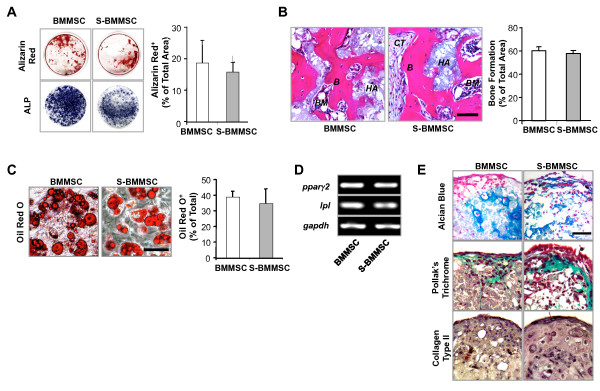
**Multipotent differentiation of S-BMMSCs**. (**A**) Alizarin Red S and alkaline phosphatase (ALP) staining showed that S-BMMSCs were similar to regular BMMSCs in osteogenic differentiation *in vitro*. (**B**) S-BMMSCs or regular BMMSCs (4 × 10^6 ^cells/transplant) were transplanted into immunocompromised mice using HA/TCP (HA) as a carrier for eight weeks. Bone formation was detected in S-BMMSC and BMMSC transplants, evidenced by H & E staining. HA, hydroxyapatite tricalcium phosphate; B, bone; M, bone marrow; CT, connective tissue. Bar = 50 μm. (**C-D**) S-BMMSCs are capable of forming Oil Red O positive cells (**C**) and expression of *pparγ*2 and *lpl *mRNA as seen in regular BMMSCs (**D**). Glyceraldehyde 3-phosphate dehydrogenase (*gapdh*) was used as an internal control. The results are representative of five independent experiments. Scale bars = 100 μm. (**E**) Chondrogenic differentiation was assessed by Alcian blue staining for acidic sulfated mucosubstances, Pollak's Trichrome staining for collagen, and immunohistochemical staining for collagen type II. S-BMMSCs were able to differentiate into chondrocytes as observed in regular BMMSCs. Bar = 50 μm. The results are representative of three independent experiments. The graph bar represents mean ± SD. BMMSCs, bone marrow mesenchymal stem cells; S-BMMSCs, BMMSCs in suspension; SD, standard deviation.

To examine the multipotent differentiation potential, we showed that S-BMMSCs are analogous to BMMSCs in their expression of alkaline phosphatase (ALP), mineralized nodule accumulation under the osteogenic inductive cultures, and bone regeneration when transplanted into immunocompromised mice using HA/TCP as a carrier (Figures 2A and 2B). Furthermore, we showed that S-BMMSCs were similar to regular BMMSCs in forming Oil red-O positive fat cells under adipogenic inductive conditions, which was associated with expression of the adipogenic genes, peroxisome proliferator-activated receptor gamma 2 (*ppar*γ2) and lipoprotein lipase (*lpl*) (Figures 2C and 2D). Parallel studies showed a similar capacity between S-BMMSCs and regular BMMSCs to differentiate into chondrocytes under chondrogenic inductive conditions, associated with the expression of proteoglycan, trichrome positive collagen, and type II collagen (Figure 2E). Collectively, these data confirm that S-BMMSCs are a subset of BMMSCs.

### S-BMMSCs express CD34, but are distinct from hematopoietic stem cells

**Figure 3:**
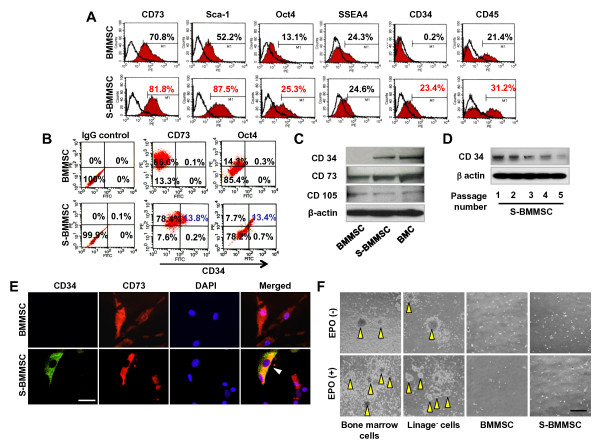
**S-BMMSCs express CD34**. (**A**) Flow cytometric analysis showed that regular BMMSCs fail to express CD34, but are positive for CD45 antibody staining (21.4%). However, S-BMMSCs express both CD34 (23.4%) and CD45 (31.2%). (**B**) Flow cytometric analysis also showed that CD34^+ ^S-BMMSCs were positive for anti CD73 (13.8%) and Oct4 (13.4%) antibody staining. IgG isotype staining groups were used as negative controls. (**C, D**) Western blot analysis indicated that S-BMMSCs express CD34 and mesenchymal surface molecules CD73 and CD105. In contrast, regular BMMSCs only express CD73 and CD105 (**C**). S-BMMSCs express CD34 at passage one to five (**D**). β-actin was used as a sample loading control. BMC, whole bone marrow ANC. (**E**) Immunocytostaining confirmed that S-BMMSCs are double positive for CD34/CD73 (triangle). Regular BMMSCs are negative for CD34 antibody staining and only positive for anti CD73 antibody staining. Bar = 100 μm. (**F**) Both BMMSCs and S-BMMSCs failed to differentiate into hematopoietic lineage under hematopoietic inductive conditions with EPO (upper panel) or without EPO (lower panel). Whole bone marrow cells and lineage negative cells were used as positive (yellow arrowheads) control. Bar = 100 μm. ANC, all nucleated cells; BMMSCs, bone marrow mesenchymal stem cells; EPO, erythropoietin; S-BMMSCs, BMMSCs in suspension.

By flow cytometric analysis, S-BMMSCs expressed mesenchymal stem cell markers at the same level as regular BMMSCs (Figure 3A). Interestingly, 23.4% of S-BMMSCs expressed CD34, a hematopoietic stem cell (HSC) and endothelial cell marker, whereas 0.2% of BMMSCs expressed CD34 (Figure 3A). BMMSCs (21.4%) and S-BMMSCs (31.2%) expressed CD45, another hematopoietic marker, at passage 2 (Figure 3A). Both BMMSCs and S-BMMSCs were negative to CD11b antibody staining (data not shown), excluding the possibility that S-BMMSCs are derived from monocyte/macrophage lineage cells. Importantly, CD34^+ ^S-BMMSCs co-expressed BMMSC-associated markers CD73 or Octamer-4 (Oct4), as evidenced by flow cytometric analysis (Figure 3B). Western blot analysis confirmed that S-BMMSCs expressed CD34, CD73, and CD105 (Figure 3C), and regular BMMSCs expressed CD73 and CD105 but lacked CD34 expression (Figure 3C). Whole bone marrow cells (BMC) were used as positive control. S-BMMSCs also showed a continued expression of CD34 from passage one to five; however, the expression levels appear reduced after passage three (Figure 3D). In order to further verify CD34 expression in S-BMMSCs, immunocytostaining analyses were performed to show co-expression of CD34 with mesenchymal markers CD73 (Figure 3E) in contrast to regular BMMSCs that were negative for anti-CD34 antibody staining (Figure 3E).

It is generally believed that CD34 expression is associated with HSCs and endothelial populations. HSCs can differentiate into all the blood cell lineages and rescue lethally irradiated subjects. Thus, we cultured S-BMMSCs and regular BMMSCs in hematopoietic differentiation medium and determined that these mesenchymal cells failed to differentiate into a hematopoietic cell lineage compare to bone marrow cells that formed myeloid and erythroid colony forming clusters (Figure 3F). In addition, CD45^-^CD34^-^BMMSCs showed an ability similar to that of S-BMMSCs in colony forming and expressing surface marker as MSC [see Additional file 1, Figure S2]. Furthermore, we infused S-BMMSCs systemically to rescue lethally irradiated mice and found that S-BMMSCs, but not regular BMMSCs, could extend the lifespan of lethally irradiated mice [see Additional file 1, Figure S3]. However, S-BMMSCs failed to rescue lethally irradiated mice, as shown in the whole bone marrow cell group [see Additional file 1, Figure S3]. These data provid further evidence that CD34 expression in S-BMMSCs is not due to HSC contamination.

### S-BMMSCs transplantation ameliorates multiple organ dysfunctions in MRL*/lpr *mice

**Figure 4:**
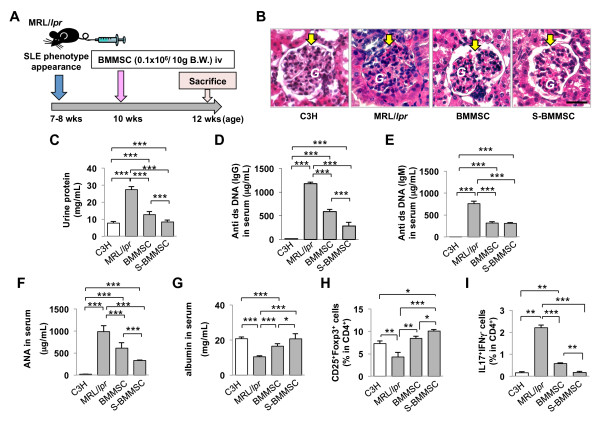
**S-BMMSCs showed superior therapeutic effect on SLE-like MRL/*lpr *mice**. (**A**) Schema of BMMSC transplantation into MRL/*lpr *mice. (**B**) S-BMMSC and BMMSC treatment recover basal membrane disorder and mesangium cell over-growth in glomerular (G) (H&E staining). (**C**) S-BMMSC and BMMSC transplantation could reduce urine protein levels at two weeks post transplantation compared to the MRL/*lpr *group. S-BMMSCs offered a more significant reduction compared to BMMSCs. (**D, E**) The serum levels of anti-dsDNA IgG and IgM antibodies were significantly increased in MRL/*lpr *mice compared to controls (C3H). S-BMMSC and BMMSC treatments could reduce antibody levels but S-BMMSCs showed a superior treatment effect than BMMSC in reducing anti-dsDNA IgG antibody (**D**). (**F**) S-BMMSC and BMMSC treatments could reduce increased levels of anti nuclear antibody (ANA) in MRL/*lpr *mice. S-BMMSC showed a better effect in ANA reduction compared to BMMSC. (**G**) S-BMMSC and BMMSC treatments could increase the albumin level in MRL/*lpr *mice, which was decreased in controls. S-BMMSC treatments were more effective in elevating the albumin level compared to BMMSC treatment. (**H**) Flow cytometric analysis showed a reduced number of Tregs in MRL/*lpr *peripheral blood compared to control. BMMSC and S-BMMSC treatments elevated the number of Tregs. S-BMMSCs induced a more significant elevation of the Tregs level than BMMSCs. (**I**) Flow cytometric analysis showed an increased number of Th17 in MRL/*lpr *mice peripheral blood compared to control. Th17 were markedly decreased in BMMSC and S-BMMSC treated groups. S-BMMSC treatment induced a more significant reduction of Th17 cells than treatment with BMMSCs. *P <0.05; ** *P *<0.01; ***P <0.001. The graph bar represents mean ± SD. BMMSCs, bone marrow mesenchymal stem cells; Ig, immunoglobulin; S-BMMSCs, BMMSCs in suspension; SD, standard deviation; SLE, systemic lupus erythematosus; Tregs, regulatory T cells.

Since the immunomodulation property of MSCs is one of the essential factors for MSC characterization, allogenic S-BMMSC transplantation into MRL/*lpr *mice was performed (Figure 4A). Two weeks after transplantation, both S-BMMSCs and BMMSCs were capable of ameliorating SLE-induced glomerular basal membrane disorder (yellow arrow, Figure 4B) and reducing the urine protein level (Figure 4C). It appeared that S-BMMSCs were superior compared to BMMSCs in terms of reducing the overall urine protein levels (Figure 4C). As expected, MRL/*lpr *mice showed remarkably increased levels of autoantibodies, including anti-double strand DNA (dsDNA) IgG and IgM antibodies (Figures 4D and 4E) and anti-nuclear antibody (ANA; Figure 4F) in the peripheral blood serum. Although S-BMMSC and BMMSC infusion showed significantly decreased serum levels of anti-dsDNA IgG, IgM antibodies and ANA in peripheral blood (Figures 4D-F), S-BMMSCs showed a superior therapeutic effect in reducing anti-dsDNA IgG antibody and ANA levels when compared to BMMSCs (Figures 4D and 4F). Additionally, decreased serum albumin levels in MRL/*lpr *mice were recovered by S-BMMSC and BMMSC infusion (Figure 4G) but S-BMMSC treatment resulted in a more significant recovery than BMMSC treatment (Figure 4G). Next, flow cytometric analysis revealed that S-BMMSC showed more effectiveness in recovering the decreased level of CD4^+^CD25^+^Foxp3^+ ^Tregs and increased the number of CD4^+^IL17^+^IFNγ^- ^T-lymphocytes (Th17 cells) in peripheral blood when compared to BMMSCs (Figures 4H, 4I). In addition, highly passaged mouse S-BMMSCs failed to inhibit Th17 differentiation *in vitro *(data not shown) suggesting that mouse S-BMMSCs probably lose their immunomodulation property under long culture expansion.

Furthermore, we showed that S-BMMSCs were superior to BMMSCs in terms of reducing increased numbers of tartrate-resistant acid phosphatase (TRAP) positive osteoclasts in the distal femur epiphysis of MRL*/lpr *mice [see Additional file 1, Figure S4A], elevated serum levels of sRANKL, a critical factor for osteoclastogenesis [see Additional file 1, Figure S4B] and bone resorption marker CTX [see Additional file 1, Figure S4C]. These data suggest that S-BMMSCs exhibit a superior therapeutic effect for SLE disorders compared to regular BMMSCs.

### S-BMMSCs possess superior immunomodulatory functions via high nitric oxide (NO) production

**Figure 5:**
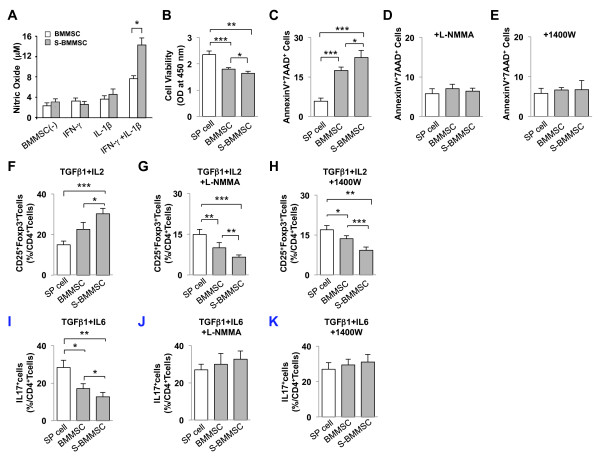
**S-BMMSCs show up-regulated immunomodulatory properties through nitric oxide (NO) production**. (**A**) NO levels in the supernatant of S-BMMSC and BMMSC culture were significantly higher in the INF-γ/IL-1β treated S-BMMSC group than in BMMSCs. (**B-C**) S-BMMSCs showed a significant reduction in the cell viability of activated SP cells compared to the cells cultured without BMMSCs (SP cell) and with BMMSCs (**B**). Both BMMSCs and S-BMMSCs showed a significantly increased rate of SP cell apoptosis compared to the SP cell only group but S-BMMSCs could induce higher SP cell apoptosis (**C**). (**D-E**) The induction of SP cell apoptosis by BMMSCs or S-BMMSCs was abolished in general NOS inhibitor L-NMMA-treated (**D) **and iNOS specific inhibitor 1400 W-treated (**E**) group. (**F-H**) Activated CD4^+^CD25^- ^T-cells and S-BMMSCs or BMMSCs were co-cultured in the presence of TGFβ1 and IL-2 with or without NOS inhibitor for three days. The floating cells were stained for CD4^+^CD25^+^FoxP3^+ ^regulatory T cells (Tregs). Both BMMSCs and S-BMMSC up-regulated Tregs but S-BMMSCs showed a significant effect in up-regulating Tregs. (**F**). Interestingly, L-NMMA and 1400 W treatments resulted in an abolishing of S-BMMSC-induced up-regulation of Tregs (**G, H**). (**I**) BMMSCs and S-BMMSCs could inhibit Th17 differentiation *in vitro*. S-BMMSC could inhibit it more effectively. (**J**, **K**) L-NMMA (**J**) or 1400 W (**K**) could abolish the inhibition of Th17 differentiation by BMMSCs or S-BMMSCs. The results are representative of at least three independent experiments. *P <0.05; **P <0.01; ***P <0.001. The graph bar represents mean ± SD. BMMSCs, bone marrow mesenchymal stem cells; iNOS, inducible nitric oxide synthase; L-NMMA, L-NG-monomethyl-arginine; NOS, nitric oxide synthase; S-BMMSCs, BMMSCs in suspension; SD, standard deviation; SP, spleen; Tregs, regulatory T cells.

Recently, immunomodulatory properties were identified as an important stem cell characteristic of BMMSCs, leading to the utilization of systemic infused BMMSCs to treat a variety of immune diseases [19-21]. Here, we found that S-BMMSCs exhibited a significantly increased capacity for NO production compared to regular BMMSCs when treated with IFNγ and IL-1β (Figure 5A). It is known that NO plays a critical role in BMMSC-mediated immunosuppression [see Additional file 1, Figures S5A-F] [29]. Therefore, we assessed the functional role of high NO production in S-BMMSC-associated immunomodulatory properties. Spleen (SP) cells were activated by anti-CD3 and anti-CD28 antibodies for three days and then co-cultured with S-BMMSCs or regular BMMSCs in the presence of the general NOS inhibitor, L-NMMA or the iNOS inhibitor, 1400 W, using a Transwell culture system. The efficacy of L-NMMA and 1400 W to inhibit NO production in BMMSCs was verified [see Additional file 1, Figures S6A and 6B]. Although both S-BMMSCs and regular BMMSCs were capable of inhibiting cell viability of activated SP cells, S-BMMSCs showed a marked inhibition of SP cell viability over that of regular BMMSCs (Figure 5B). Moreover, both BMMSCs and S-BMMSCs induced SP cell apoptosis (Figure 5C). However, S-BMMSCs showed an elevated capacity in inducing activated SP cell apoptosis compared to regular BMMSCs (Figure 5C). Interestingly, when L-NMMA and 1400 W were added to the cultures, the number of apoptotic SP cells was significantly reduced in both S-BMMSC and regular BMMSC groups (Figure 5D and 5E). These *in vitro *experimental data suggested that NO production is an essential factor for BMMSC-mediated immunomodulation.

Since up-regulation of CD4^+^CD25^+^Foxp3^+ ^Tregs is required for immunotolerance [30], we tested Tregs up-regulation property of S-BMMSCs and BMMSCs in an *in vitro *co-culture system. When naïve^-^T-cells were co-cultured with S-BMMSCs or regular BMMSCs in the presence of IL-2 and TGF-β1, S-BMMSCs showed a significant up-regulation of Treg levels compared to regular BMMSCs (Figure 5F). Both L-NMMA and 1400 W were able to inhibit BMMSC- and S-BMMSC-induced up-regulation of Tregs, as shown by flow cytometric analysis (Figures 5G and 5H). Interestingly, the regulation effect on Tregs was more significant in the S-BMMSC group compared to the BMMSC group (Figure 5G and 5H). Moreover, both BMMSCs and S-BMMSCs could inhibit differentiation of Th17 *in vitro*, with a more prominent effect observed with S-BMMSC (Figure 5I). These inhibitions of Th17 differentiation were abolished by L-NMMA (Figure 5J) and 1400 W (Figure 5K). These data further verified the functional role of NO in S-BMMSC-induced immunomodulatory effect.

In order to identify whether there are functional endogenous S-BMMSCs, we used fluorescence activated cell sorting (FACS) to isolate CD34 and CD73 double-positive cells from bone marrow ANCs which resulted in the recovery of 3.77% double-positive cells [see Additional file 1, Figure S7A]. These CD34 and CD73 double-positive cells exhibited mesenchymal stem cell characteristics, including the capacity to form single colony clusters of fibroblast-like cells [see Additional file 1, Figure S7B], which could differentiate into osteogenic cells *in vitro *[see Additional file 1, Figure S7C]. These data indicated the feasibility of this approach to isolate S-BMMSC-like cells directly from bone marrow. We found that CD34^+^/CD73^+ ^BMMSCs were analogous to S-BMMSCs in terms of having higher levels of NO production when compared to regular BMMSCs [see Additional file 1, Figure S7D] and reducing levels of urine protein, serum anti-dsDNA IgG and IgM antibodies in MRL/*lpr *mice (data not shown). These data indicate that endogenous S-BMMSCs could be isolated from bone marrow using CD34 and CD73 antibodies double sorting.

Additionally, we used the same BMMSC-ECM isolation approach to reveal the existence of human S-BMMSCs (hS-BMMSC) that possess stem cell properties including multipotent differentiation and self-renewal but lack expression of CD34 (data not shown). hS-BMMSCs showed elevated NO and kynurenine production which indicate high indoleamine 2,3-dioxygenase (IDO) activity when compared to regular BMMSCs [see Additional file 1, Figures S8A-C]. Thus, when activated T cells were co-cultured with hS-BMMSCs, AnnexinV-7 aminoactinomycinD (7AAD) double positive apoptotic SP cells were significantly elevated compared to BMMSCs [see Additional file 1, Figure S8D].

### Discussion

Adherent BMMSCs are able to proliferate and undergo osteogenic differentiation, providing the first evidence of CFU-F as precursors for osteoblastic lineage [25]. For over a few decades, the adherent CFU-F assay has been used as an effective approach to identify and select BMMSCs. In the current study, we showed that the adherent CFU-F assay collects the majority of clonogenic BMMSCs, but a subpopulation of BMMSCs is sustained in the culture suspension. This newly identified subpopulation of BMMSCs may be lost in the standard CFU-F assay for BMMSC isolation.

Due to the heterogeneity of the BMMSCs, there is no single, unique marker allowing for BMMSC isolation, rather an array of cell molecules are utilized to profile BMMSCs. It is widely accepted that BMMSCs express SH2 (CD105), SH3/SH4 (CD73), integrin β_1 _(CD29), CD44, Thy-1 (CD90), CD71, vascular cell adhesion molecule-1 (CD106), activated leukocyte cell adhesion molecule (CD166), STRO-1, GD2, and melanoma cell adhesion molecule (CD146) [5,7-13,31,32]. Nevertheless, it is believed that BMMSCs lack expression of hematopoietic surface molecules including CD34, integrin α_M _(CD11b) and CD14. However, recent studies have implied that mouse BMMSCs might express the hematopoietic surface molecules, CD45 [28] and CD34 [33]. To ensure purity of S-BMMSCs, we used immune FACS to collect SSEA4^+ ^S-BMMSCs for proliferation and differentiation assays in this study. Interestingly, previous experimental evidence appeared to support a notion that HSCs are capable of differentiating into mesenchymal cells [34] and osteoblastic lineage *in vivo *[35]. Thus, it is critical to clarify whether BMMSCs express hematopoietic associated surface molecules.

In this study, we have identified a novel subset of S-BMMSCs that failed to form adherent CFU-F in regular culture dishes, but were capable of adhering on mesenchymal stem cell-produced ECM and differentiating into osteoblasts, adipocytes and chondrocytes from both C3H/HeJ and C57BL/6J mice. S-BMMSCs co-expressed the HSC marker CD34 with the MSC markers CD73 and Oct4, excluding the potential of HSC contamination. Furthermore, S-BMMSCs were found to be distinct from HSC because they lacked the ability to differentiate into hematopoietic cell lineages *in vitro *and failed to rescue lethally-irradiated mice. The mechanism that may contribute to the up-regulated immunomodulatory function was associated with high NO production in S-BMMSCs and a NO-driven high Tregs level [36]. NO is a gaseous biological mediator with important roles in affecting T cell function [37].

This is the reason that S-BMMSCs showed a superior therapeutic effect in treating SLE mice.

One successful approach is to isolate cells that express specific molecules on their cell surfaces using monoclonal antibodies and cell sorting technologies. Enriched populations of BMMSCs have been isolated from human bone marrow aspirates using a STRO-1 monoclonal antibody in conjunction with antibodies against VCAM-1/CD106 [32], CD146 [11], low affinity nerve growth factor receptor/CD271, PDGR-R, EGF-R and IGF-1-R [38], fibroblast cell marker/D7-Fib [39] and integrin alpha 1/CD49a [40]. A more recent study has also identified molecules co-expressed by a CD271^+ ^mesenchymal stem cell population including platelet derived growth factor receptor-β (CD140b), human epidermal growth factor 2/ErbB2 (CD340) and frizzled-9 (CD349) [41]. Further cell separation based upon multi-parameter FACS identified a population of proposed mouse mesenchymal precursors with the composite phenotype Lin^-^CD45^-^CD31^-^Sca-1^+ ^[42]. Another recent study also identified and characterized an alternate population of primitive mesenchymal cells derived from adult mouse bone marrow, based upon their expression of the SSEA-1 [43]. All approaches used for BMMSC purification and isolation will undergo *ex vivo *expansion to enrich cell numbers for tissue regeneration or systemic therapies by plastic adherent assay. In addition to identifying a novel sub-population of BMMSCs that possess enhanced immunomodulatory properties when compared to regular BMMSCs, we showed that CD34^+^/CD73^+ ^BMMSCs could be isolated directly from whole bone marrow and that CD34^+^/CD73^+ ^BMMSCs are endogenous S-BMMSCs with higher NO production, and are superior in treating SLE-like mice when compared to regular BMMSCs.

Recently, non-adherent bone marrow cells (NA-BMCs) were identified [44,45]. The NA-BMSCs could be expanded in suspension and gave rise to multiple mesenchymal phenotypes, including osteoblasts, chondrocytes, and adipocytes *in vitro*, suggesting the presence of non-adherent BMMSCs in primary CFU-F cultures [45]. Although it has been reported that the NA-BMCs can rescue lethally-irradiated mouse recipients, our data indicated that S-BMMSCs only showed improved survival lifespan without a complete rescue of lethally-irradiated mice, compared to whole bone marrow transplantation. While the mechanism of S-BMMSC-mediated lifespan extension in lethally-irradiated mice is unknown, it is possible that S-BMMSCs have a more active interplay with hematopoietic cells than regular BMMSCs. It has been reported that granulocyte colony stimulating factor might promote BMMSCs into the circulation in humans [46], suggesting that non-attached BMMSCs may exist *in vivo *for specific functional needs. Added evidence indicated that osteocalcin-positive cells in circulation were able to differentiate into osteoblastic cells when cultured in the presence of TGFβ [47]. However, it is unknown whether S-BMMSCs are associated with circulating mesenchymal stem cells initially identified in mice, and this is very rare in humans.

## Conclusions

A new subset of BMMSCs (S-BMMSCs) which failed to adhere to culture dishes possesses similar stem cell properties as those seen in BMMSCs, including CFU-F, stem cell markers, osto-, adipo-, and chondro-genic differentiation. However, S-BMMSC showed distinct features including expression of CD34 and a superior immunomodulation property through high NO production. These findings suggest that it is feasible to improve immunotherapy by identifying new subset BMMSCs.

## Abbreviations

7AAD: 7aminoactinomycineD; ALP: alkaline phosphatase; ANCs: all nucleated cells; BMMSCs: bone marrow mesenchymal stem cells; BrdU: bromodeoxyuridine; CFU-F: colony forming unit fibroblastic; CTX: C-terminal telopeptides of type I collagen; DAPI: 4', 6-diamidino-2-phenylindole; (D)MEM: (Dulbecco's) modified Eagle's medium; ECM: extracellular cell matrix; ELISA: enzyme-linked immunosorbent assay; EPO: erythropoietin; FACS: fluorescence-activated cell sorting; FBS: fetal bovine serums; FITC: fluorescein isothiocyanate; H & E: hematoxylin and eosin; HA/TCP: hydroxyapatite/tricalcium phosphate; HSC: hematopoietic stem cell; IDO: indoleamine 2,3-dioxygenase; IFNγ: interferon gamma; IgG: immunoglobulin G; IL-1β: interleukin-1 beta; iNOS: inducible NOS; L-NMMA: L-NG-monomethyl-arginine; lpl: lipoprotein lipase; NF-κB: nuclear factor-kappa B; NOS: nitric oxide synthase; PBMNCs: peripheral blood mononuclear cells; PBS: phosphate-buffered saline; PE: phycoerythrin; PFA: paraformaldehyde; pparγ2: peroxisome proliferator-activated receptor gamma 2; RT-PCR: reverse transcriptase polymerase chain reaction; S-BMMSC: BMMSCs in suspension; SLE: systemic lupus erythematosus; SP: spleen; sRANKL: soluble runt-related NF-κB ligand; SSEA: stage-specific embryonic antigen; TGFβ: transforming growth factor beta; Th17: T helper 17 cells; TRAP: tartrate-resistant acid phosphatase; Tregs: regulatory T cells.

## Competing interests

The authors declare that they have no competing interests.

## Authors' contributions

KA and YY: contributions to conception and design of experiments, acquisition of data, analysis and interpretation of data. TY, CC, LT, and YJ: contributions to acquisition of data, analysis and interpretation of data. XC and SG: contributions to drafting the manuscript and revising critically. SS: contributions to conception and design, drafting the manuscript, and giving final approval of the version to be published. All authors have read and approved the manuscript for publication.

## Supplementary Material

Additional file 1 **Figures S1 to S8 and Additional materials and methods. Figure S1**. **ECM coated dish could capture a greater number of CFU-F**. CFU-f number in ECM coated dish compared to regular dish. **Figure S2**. **CD45^-^CD34^-^BMMSCs showed similar property with S-BMMSCs**. (A) CFU-f number. (B) Flow cytometric analysis. **Figure S3. S-BMMSCs extended survival rate of lethal dose of irradiated mice**. The life span of irradiated mice. **Figure S4. Osteoclast activity in S-BMMSC-treated *MRL/lpr *mice**. (A) Osteoclast number. (B) sRANKL level. (C) CTX level. **Figure S5. L-NMMA pre-treated BMMSC transplantation failed to ameliorate disease phenotype of *MRL/lpr *mice**. (A) Anti dsDNA (IgG) level. (B) Anti dsDNA (IgM) level. (C) Urine protein level. (D) Tregs level. (E) Th17 level. (F) Ratio between Tregs/Th17. **Figure S6. Inhibition of NO production in BMMSCs**. (A) NO level with inhibitors. (B) iNOS level by western blot. **Figure S7. Endogenous S-BMMSCs in mice bone marrow**. (A) Cell sorting result. (B) CFU-f number. (C) Osteogenic differentiation *in vitro*. (D) NO level. **Figure S8. Human bone marrow contains S-BMMSCs (hS-BMMSCs)**. (A) NO level. (B) Kynurenine production. (C) Kynurenine production in co-culture system. (D) T cell apoptosis induction by hS-BMMSCs. Additional materials and methods describe about TRAP staining, Histomotry, Rescue lethal dose irradiated mice, and Isolation of CD34^+^CD73^+ ^double positive cells. Click here for file
