# Supplementary material for: Characterization of bone marrow derived mesenchymal stem cells in suspension
Source: Stem Cell Res Ther. 2012 Oct 19;3(5):40. doi: 10.1186/scrt131 (PMC3580431; doi:10.1186/scrt131)
Supplement: Additional file 1 — Figures S1 to S8 and Additional materials and methods. Figure S1. ECM coated dish could capture a greater number of CFU-F. CFU-f number in ECM coated dish compared to regular dish. Figure S2. CD45-CD34-BMMSCs showed similar property with S-BMMSCs. (A) CFU-f number. (B) Flow cytometric analysis. Figure S3. S-BMMSCs extended survival rate of lethal dose of irradiated mice. The life span of irradiated mice. Figure S4. Osteoclast activity in S-BMMSC-treated MRL/lpr mice. (A) Osteoclast number. (B) sRANKL level. (C) CTX level. Figure S5. L-NMMA pre-treated BMMSC transplantation failed to ameliorate disease phenotype of MRL/lpr mice. (A) Anti dsDNA (IgG) level. (B) Anti dsDNA (IgM) level. (C) Urine protein level. (D) Tregs level. (E) Th17 level. (F) Ratio between Tregs/Th17. Figure S6. Inhibition of NO production in BMMSCs. (A) NO level with inhibitors. (B) iNOS level by western blot. Figure S7. Endogenous S-BMMSCs in mice bone marrow. (A) Cell sorting result. (B) CFU-f number. (C) Osteogenic differentiation in vitro. (D) NO level. Figure S8. Human bone marrow contains S-BMMSCs (hS-BMMSCs). (A) NO level. (B) Kynurenine production. (C) Kynurenine production in co-culture system. (D) T cell apoptosis induction by hS-BMMSCs. Additional materials and methods describe about TRAP staining, Histomotry, Rescue lethal dose irradiated mice, and Isolation of CD34+CD73+ double positive cells. [file scrt131-S1.DOC]

Additional file 1 for

Characterization of Bone Marrow Derived Mesenchymal Stem Cells

in Suspension.

Kentaro Akiyama^1, 2*^, Yong-Ouk You ^1*^, Takayoshi Yamaza ^1, 3^, Chider Chen^1^, Liang Tang^4^, Yan Jin^4^, Xiao-Dong Chen^5^, Stan Gronthos^6^, Songtao Shi^1^.

^1^Center for Craniofacial Molecular Biology, University of Southern California, 2250 Alcazar Street, CSA 103, Los Angeles, CA 90033, USA

^2^Department of Oral Rehabilitation and Regenerative Medicine, Okayama University Graduate School of Medicine, Dentistry, and Pharmaceutical science, 2-5-1 Shikata-cho, Kita-ku, Okayama 700-8525, Japan

^3^Department of Molecular Cell Biology and Oral Anatomy, Kyushu University Graduate School of Dental Science, Fukuoka 812-8582, Japan

^4^Research and Development Center for Tissue Engineering, Fourth Military Medical University, Xi'an, Shanxi, China.

^5^University of Texas Health Science Center, School of Dentistry, San Antonio, Texas 78245, USA

^6^Mesenchymal Stem Cell Group, Department of Haematology, Institute of Medical and Veterinary Science/ Hanson Institute, Adelaide 5000, South Australia, Australia

**
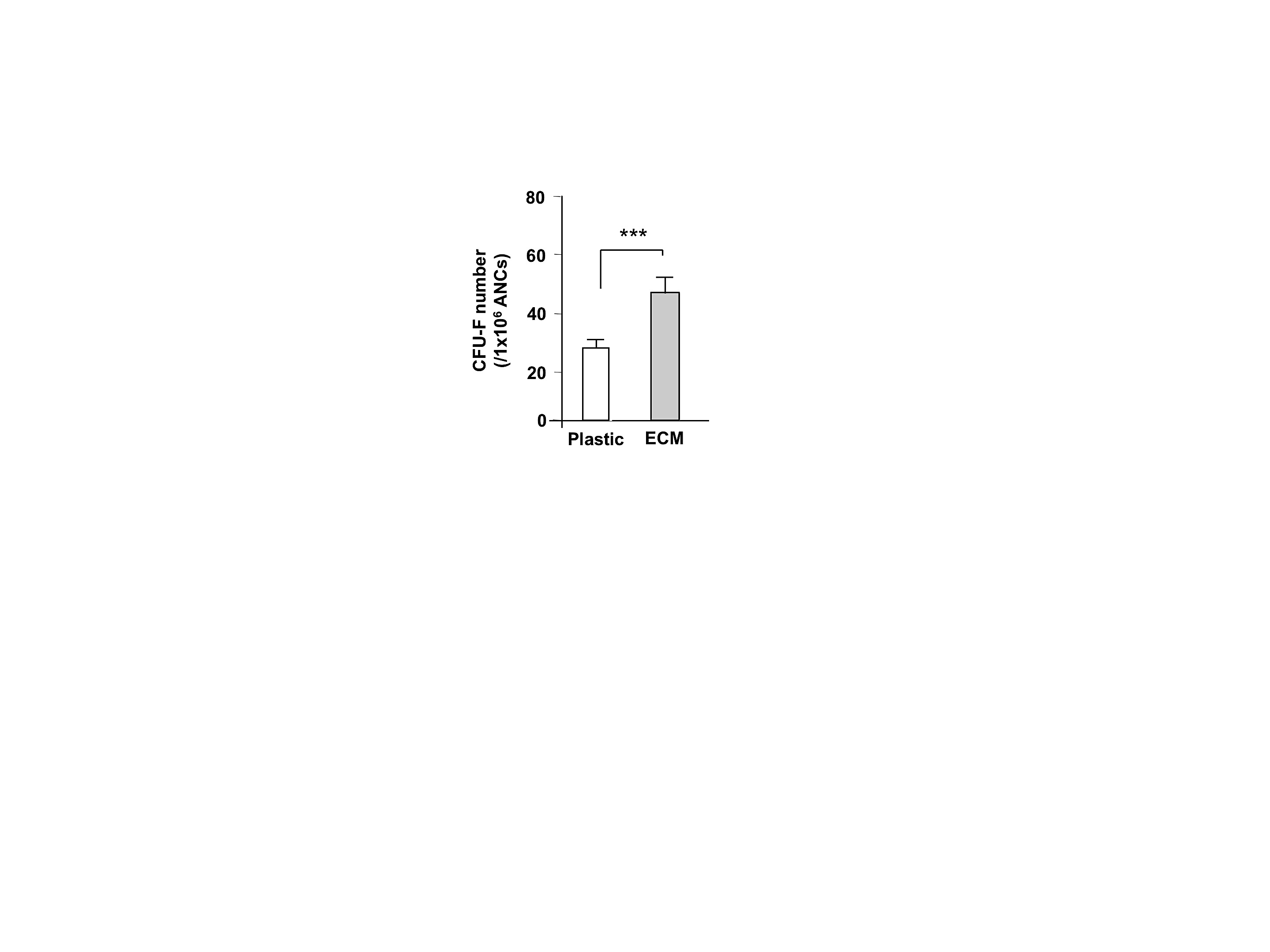
**

**Figure S1**. **ECM coated dish could capture grater number of CFU-F**. Primary ANCs were seeded at 1x10^6^ into 60mm normal plastic culture dishes (Plastic) or the ECM which was produced by BMMSCs coated culture dishes (ECM) for 14 days. The CFU-F number was significantly increased in BMMSCs cultured in ECM coated dishes. The results were representative of five independent experiments. ****P*<0.005. The graph bar represents mean±SD.

**Figure S2**. **CD45^-^CD34^-^BMMSCs showed similar property with S-BMMSCs.**  (**A-B**) CD45^-^CD34^-^BMMSCs were sorted and compared to S-BMMSC in colony forming (**A**) and surface marker analysis (**B**). There are no significant differences between these cells in CFU-f and flow cytometric analysis. The graph bar represents mean±SD.


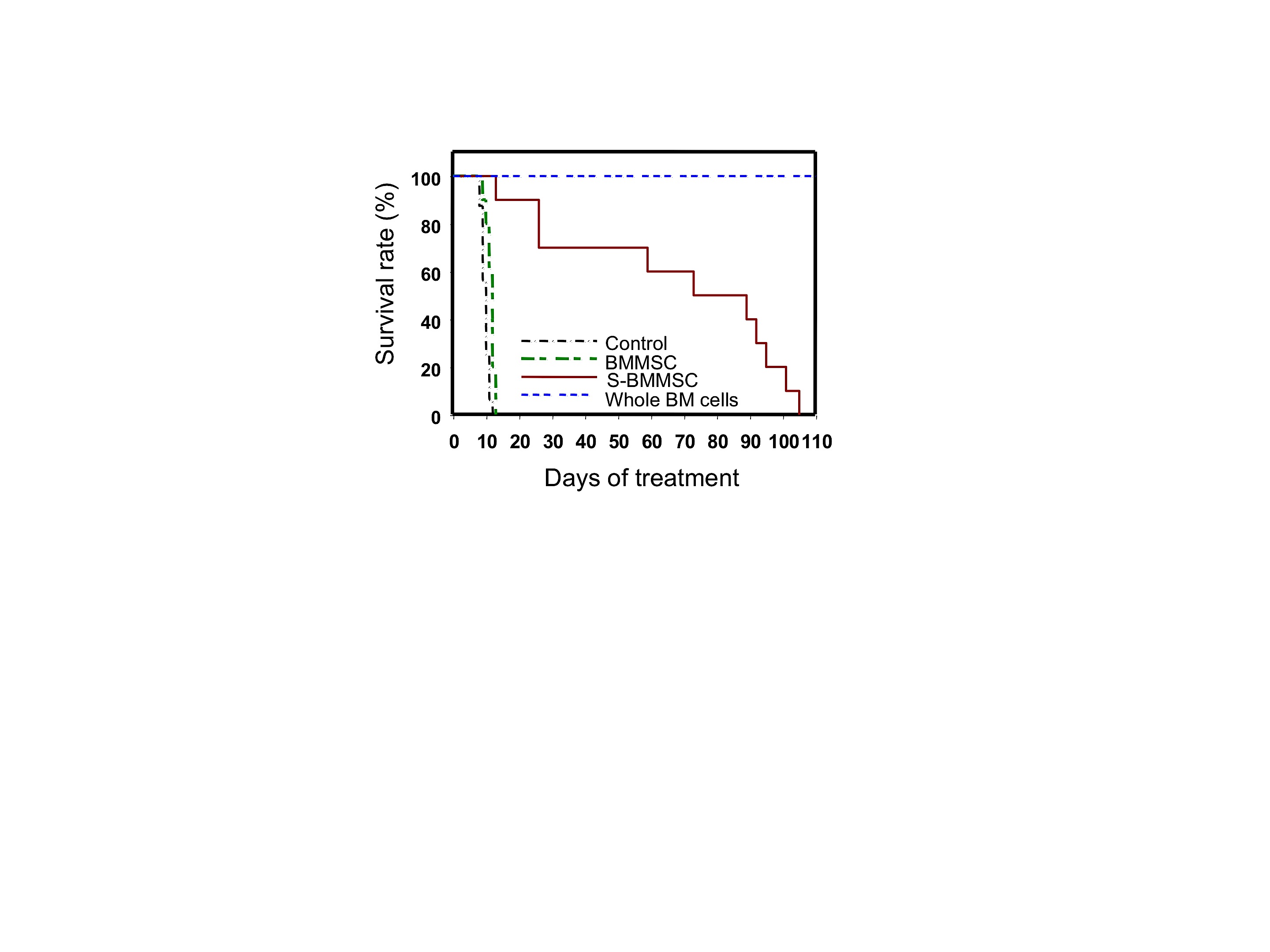
 **Figure S3**.  **S-BMMSCs extended survival rate of lethal dose of irradiated mice.** Mice received either regular BMMSCs (BMMSC, n=5) or PBS without cells (Control, n=8) failed to survive over 14 days. The whole bone marrow cell infusion group (Whole BM cells, n=3) is a positive control group with survival over 110 days after irradiation. S-BMMSCs can extend life span of lethal dose irradiated mice (S-BMMSC, n=10). Kaplan-meier survival curves.


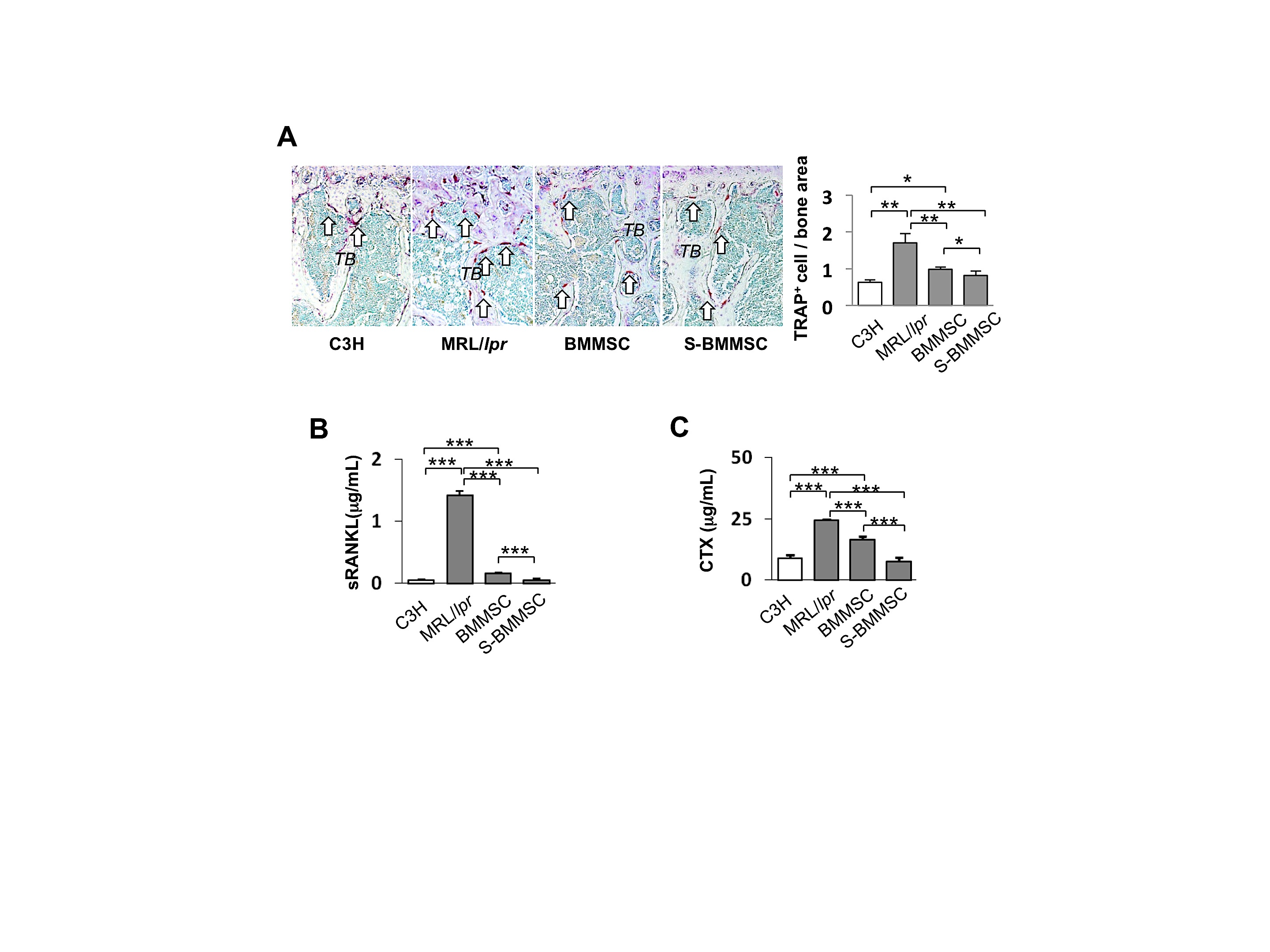


**Figure S4**. **Osteoclast activity in S-BMMSC-treated *MRL/lpr* mice.** (**A**) TRAP staining indicated the increased number of TRAP positive cells in epiphysis of the distal femurs of MRL/*lpr* mice as compared to the control (C3H). S-BMMSC and BMMSC infusion resulted in a significant reduced number of TRAP positive cells. It appears that S-BMMSC group shows more significant reduction of number of TRAP positive cells than BMMSC group. (**B**, **C**) ELISA revealed that MRL/*lpr* mice have increased levels of soluble RANKL (sRANKL) (**B**) and C-terminal telopeptides of type I collagen (CTX) (**C**) in serum as compared to the controls. S-BMMSC and BMMSC infusion can significantly reduce levels of sRANKL (**B**) and CTX (**C**), but S-BMMSC group showed a more effective in reduce levels of sRANKL (**B**) and CTX (**C**). The results were representative of five independent experiments. **P*<0.05; ***P*<0.01; ****P*<0.005. The graph bar represents mean±SD.


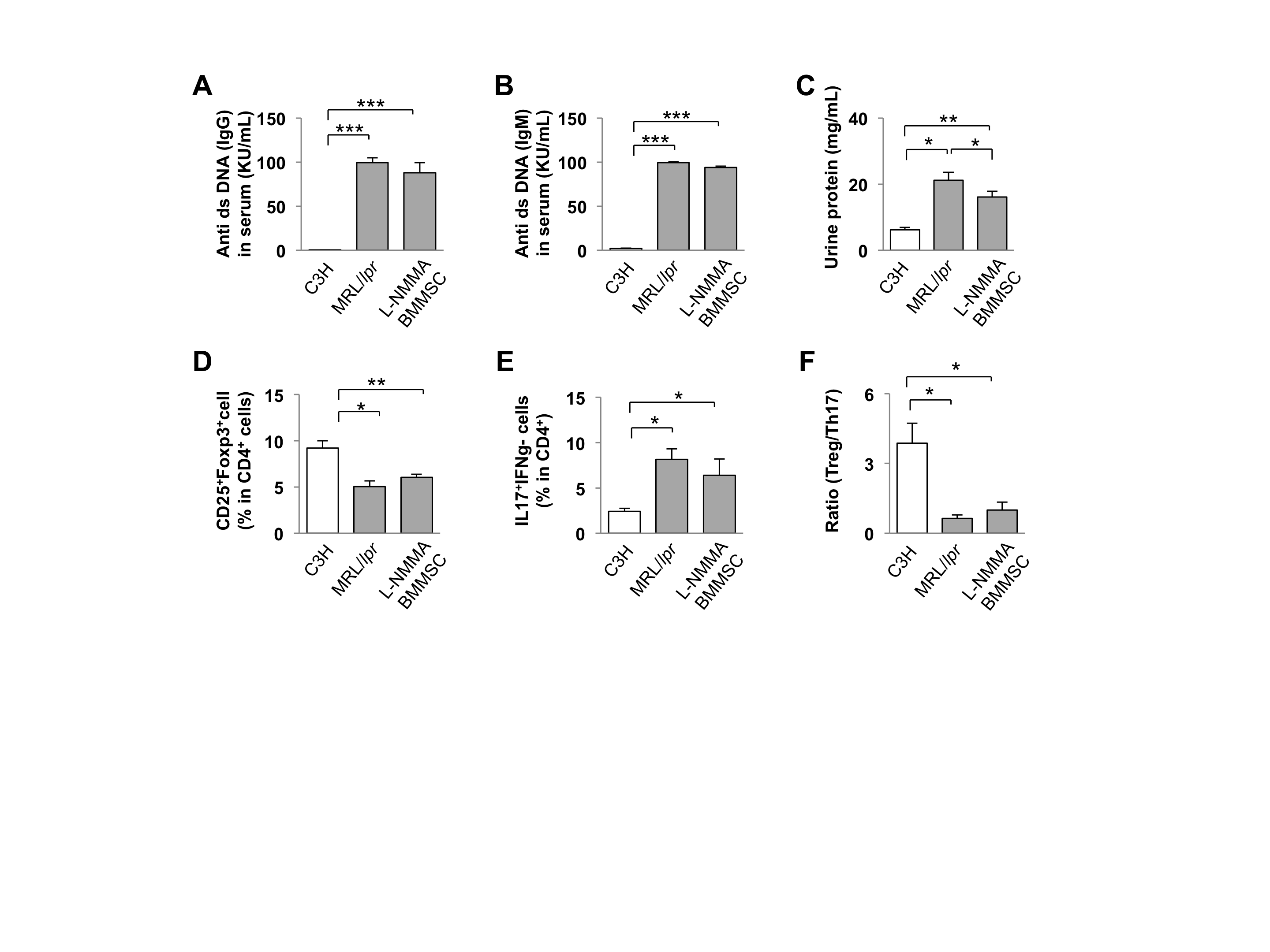


**Figure S5**. **L-NMMA pre-treated BMMSC transplantation failed to ameliorate disease phenotype of *MRL/lpr* mice.** (**A-B**) L-NMMA pre-treated BMMSCs (n=5) failed to reduce increased level of anti ds DNA IgG (**A**) and IgM (**B**) in MRL/*lpr* mice. (**C**) L-NMMA pre-treated BMMSC could reduce urine protein level in MRL/*lpr* mice but still not same level as control (n=4). (**D**) Decreased level of CD25^+^Foxp3^+^Tregs in MRL/*lpr* mice was not recovered after L-NMMA treated BMMSC transplantation. (**E**) L-NMMA pre-treated BMMSC failed to reduce CD4^+^IL17^+^Th17 cells in MRL/*lpr* mice. (**F**) The ratio of Treg/Th17 in MRL/lpr mice was not improved after L-NMMA treated BMMSC transplantation. **P*<0.05; ***P*<0.01; ****P*<0.005. The graph bar represents mean±SD.

**
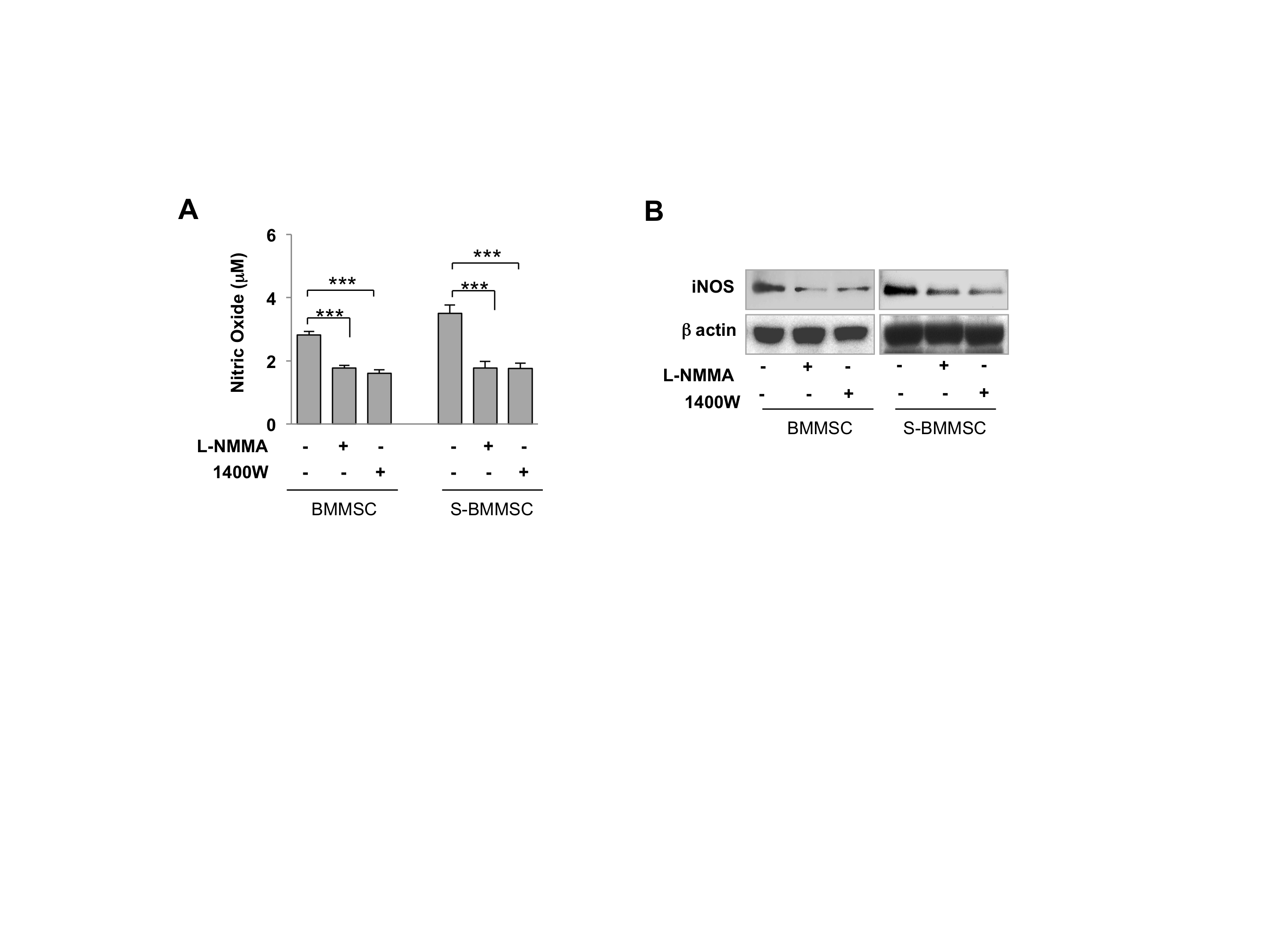
**

**Figure S6**. **Inhibition of NO production in BMMSCs.** BMMSCs and S-BMMSCs (2x10^5^/well) were cultured for 3 days and treated with L-NMMA (1 mM) or 1400W (0.2 mM) for 3days. (**A**) NO level from in culture supernatant was decreased after both L-NMMA or 1400W treatment. The results were representative of five independent experiments. (**B**) Western blot analysis showed that iNOS expression was inhibited by L-NMMA and 1400W. **P*<0.05; ****P*<0.001. The graph bar represents mean±SD.


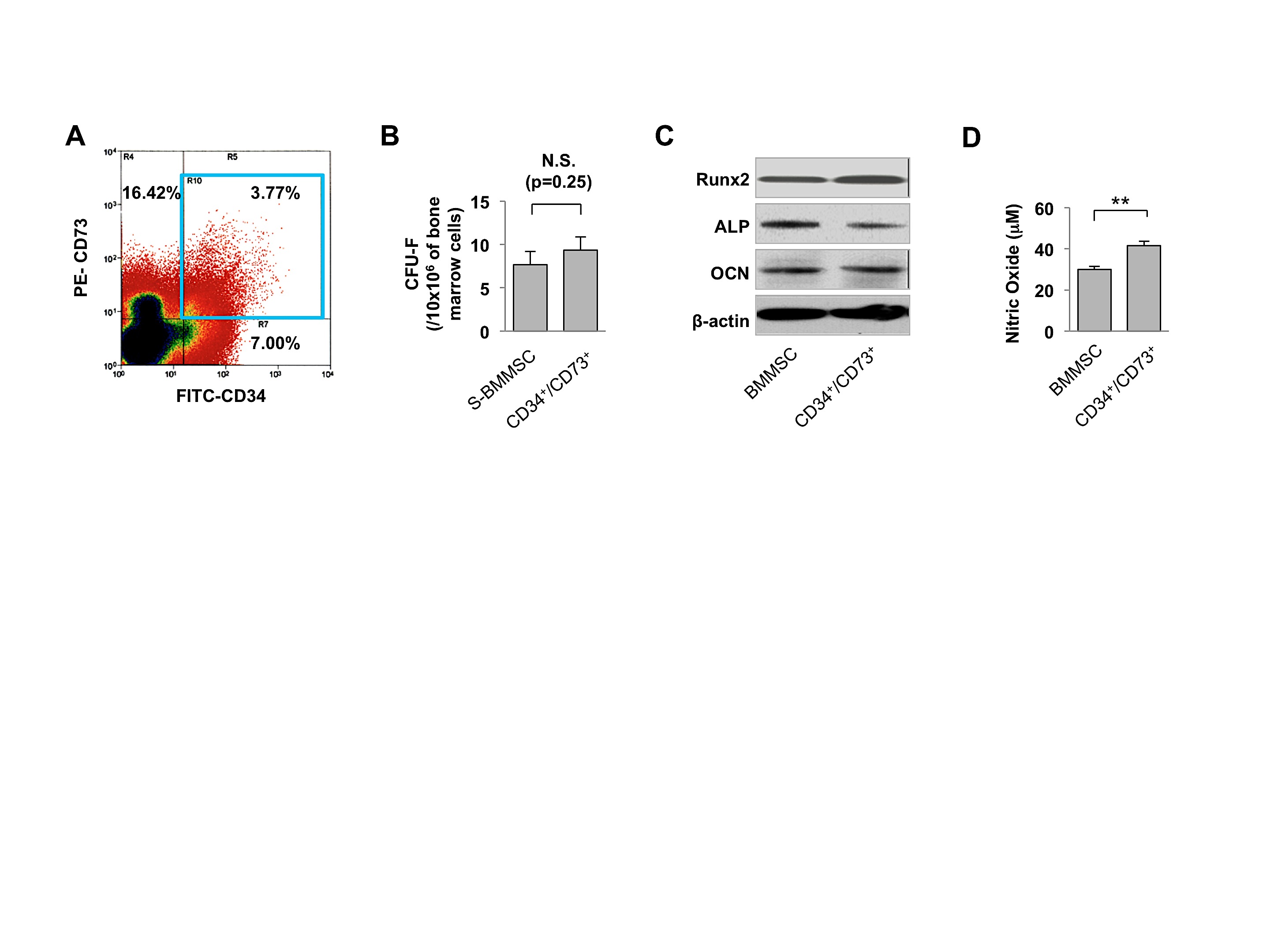


**Figure S7**. **Endogenous S-BMMSCs in mice bone marrow.** (**A**) Result of flow cytometric cell sorting. There are 3.77% cells are double positive for anti CD34 and CD73 antibody staining in whole bone marrow ANCs. (**B**) CD34^+^/CD73^+^ cells form CFU-F on BMMSC-ECM cultures at frequency similar to S-BMMSCs. (**C**) CD34^+^/CD73^+^ BMMSCs have similar osteogenic differentiation ability with BMMSCs. (**D**) CD34^+^/CD73^+^ BMMSCs also show a significant higher NO production when compared to regular BMMSCs. ***P*<0.01. The graph bar represents mean±SD.


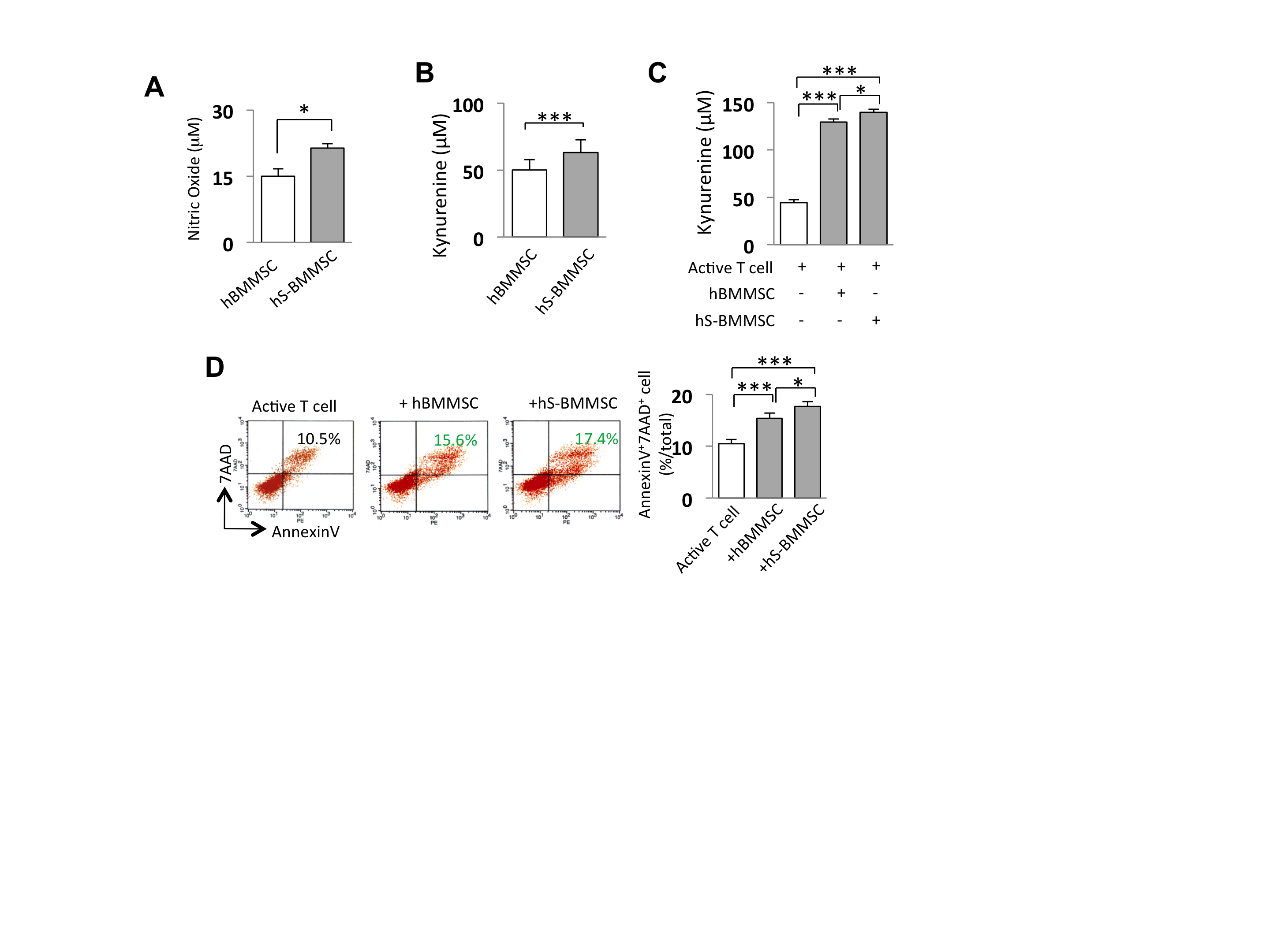


**Figure S8**. **Human bone marrow contains S-BMMSCs (hS-BMMSC).** (A) hS-BMMSCs produce high level of NO than that of hBMMSCs as assessed by Total NO/Nitrite/Nitrate kit. (B) Kynurenine production was significantly increased in hS-BMMSC compare to hBMMSC (p<0.005). (C) When hBMMSC or hS-BMMSC were co-cultured with active T cell, the kynurenine level in co-culture system was dramatically increased with more significantly increase in hS-BMMSC group compare to hBMMSC group. (D) Annexin V and 7AAD double positive apoptotic cell numbers in active T cells were increased when co-cultured with hBMMSC or hS-BMMSC. However, apoptotic cell rate was significantly increased in hS-BMMSC group compared to hBMMSC group. The results were representative of three independent experiments. **P*<0.05; ***P*<0.01; ***p<0.005. The graph bar represents mean±SD

**Additional materials and methods**

***TRAP staining***

Deparaffinized sections were re-fixed with a mixture of 50% ethanol and 50% acetone for 10 min. TRAP-staining solutions were freshly made (1.6% naphthol AS-BI phosphate in N, N-dimethylformamide and 0.14% fast red-violet LB diazonium salt, 0.097% tartaric acid and 0.04% MgCl_2_ in 0.2 M sodium acetate buffer, pH 5.0) and mixed in 1:10. The sections were incubated in the solution for 10 min at 37^o^C under shield and counterstained with toluidine blue. All regents for TRAP staining were purchased from Sigma.

***Histometry***

Area of trabecular bone was measured on bone sections with H&E staining. To quantify osteoclast activity in the bones, number of mature osteoclasts was determined by TRAP positive cells attached on the bone surface. Each number of cells and area were measured from five representative images per each sample using an NIH Image-J, followed by calculating the means. The data were average the means in each experimental group. The results were shown as each indicated percentage.

***Rescue lethal dose irradiated mice***

In each group, 1x10^6^ cells in 50 μl PBS or PBS alone as control were injected into the tail vein of recipient mice at 1 day post lethal irradiation (8.5 Gy per mouse). The survival date of each mouse was recorded and analyzed.

***Isolation of CD34^+^CD73^+^ double positive cells***

After removing red blood cells using ACK lysing buffer, bone marrow derived ANCs were stained with anti CD34-FITC and anti CD73-PE antibodies for 30 min on ice under dark condition. After wash with PBS, cells were re-suspended into OPTI-MEM (Invitrogen) supplement with 2% FBS and antibiotics (100 U/ml penicillin and 100 μg/ml streptomycin) and sorted by MOFLO XDP Cell Sorter (BECKMAN Coulter). The sorted double positive cells were seeded on ECM coated 60 mm dish at density of 1x10^6^/dish and cultured for further experiments.
